# Supplementary material for: Mitochondrial Genomes of the Blood Flukes Cardicola forsteri and Cardicola orientalis (Trematoda: Aporocotylidae)
Source: Pathogens. 2025 Jul 10;14(7):680. doi: 10.3390/pathogens14070680 (PMC12298837; doi:10.3390/pathogens14070680)
Supplement: Supplementary file 1 [file pathogens-14-00680-s001.zip › pathogens-3727687-supplementary.pdf]

Table S1. NCBI blast search of Trematode cox1 sequences.

Species	Ascension
Aporocotylidae sp.	OR030100
Austrobilharzia varglandis	AY157196
Bilharziella polonica	AY157186
Cardicola forsteri	KP988302
Cardicola opisthorchis	KP988305
Marinabilharzia patagonense	OK338769
Paradeontacylix balearicus	AM489604
Paradeontacylix godfreyi	AM489607
Paradeontacylix grandispinus	AM489606
Paradeontacylix humboldti	MW598468
Paradeontacylix ibericus	AM489603
Paradeontacylix kampachi	AM489605
Paradeontacylix olivai	MW598469
Schisosoma incognitum	JQ408708
Schistosoma bovis	PP654254
Schistosoma edwardiense	AY197347
Schistosoma hippopotami	AY197346
Schistosoma japonicum	ON637113
Schistosoma mattheei	AP017710
Schistosoma mekongi	AY157199
Schistosomatidae sp.	MG707172
Trichobilharzia anseri	KP901380
Trichobilharzia sp.	JQ681535
Trichobilharzia szidati	JF838198
Aporocotylidae sp.	OR030100

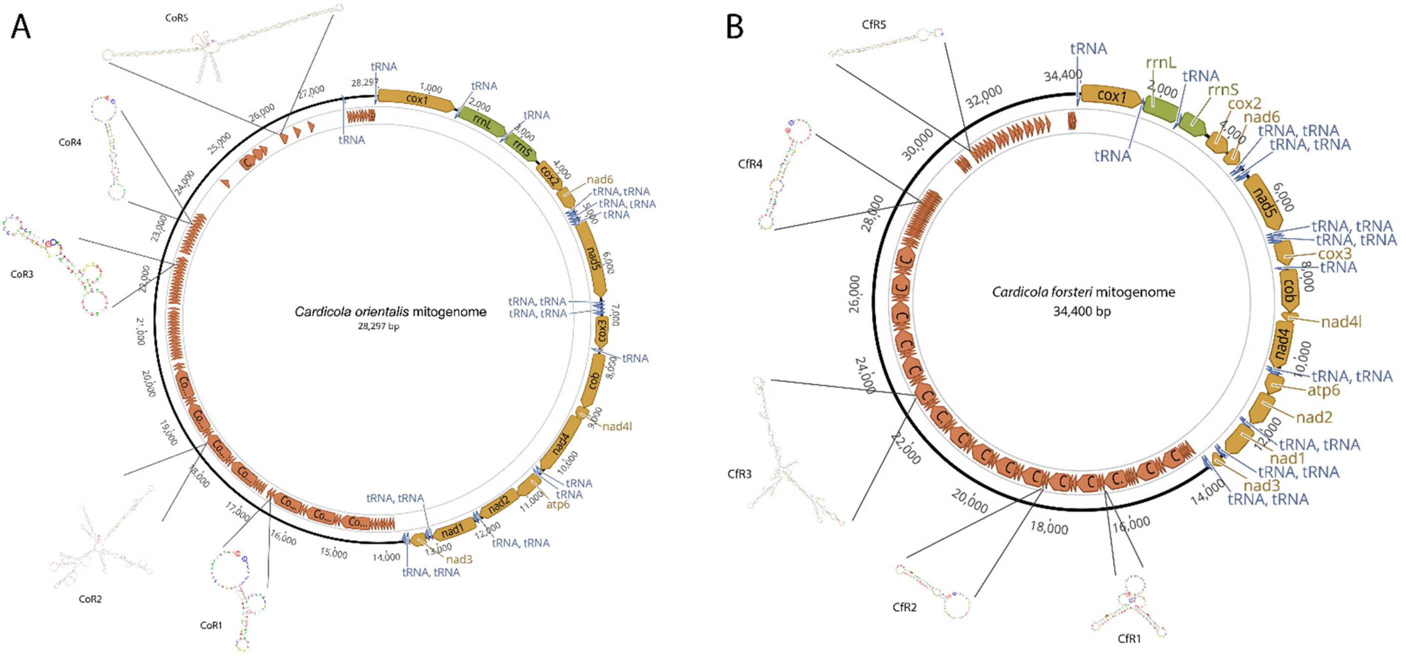

Figure S1. Circular mitochondrial genomes of (A). *C. forsteri*, and (B). *C. orientalis*, with their repeat structures.
